# Improving screening for malnourished children at high risk of death: a study of children aged 6–59 months in rural Senegal

**DOI:** 10.1017/S136898001800318X

**Published:** 2018-12-03

**Authors:** Mark Myatt, Tanya Khara, Carmel Dolan, Michel Garenne, André Briend

**Affiliations:** 1 Brixton Health, Cilfach Greigiog, Fford Celynin, Llwyngwril, Gwynedd, LL37 2JD, UK; 2 Emergency Nutrition Network, Oxford, UK; 3 IRD, UMI Résiliences, Paris, France; 4 Institut Pasteur, Epidémiologie des Maladies Emergentes, Paris, France; 5 FERDI, Université d’Auvergne, Clermont-Ferrand, France; 6 MRC/Wits Rural Public Health and Health Transitions Research Unit, School of Public Health, Faculty of Health Sciences, University of the Witwatersrand, Johannesburg, South Africa; 7 School of Medicine, Center for Child Health Research, University of Tampere, Tampere, Finland; 8 Department of Nutrition, Exercise and Sports, University of Copenhagen, Copenhagen, Denmark

**Keywords:** Wasting, Stunting, Underweight, Mid-upper arm circumference, Anthropometry, Mortality, Therapeutic feeding, Child survival

## Abstract

**Objective:**

To investigate whether children with concurrent wasting and stunting require therapeutic feeding and to better understand whether multiple diagnostic criteria are needed to identify children with a high risk of death and in need of treatment.

**Design:**

Community-based cohort study, following 5751 children through time. Each child was visited up to four times at 6-month intervals. Anthropometric measurements were taken at each visit. Survival was monitored using a demographic surveillance system operating in the study villages.

**Setting:**

Niakhar, a rural area of the Fatick region of central Senegal.

**Participants:**

Children aged 6–59 months living in thirty villages in the study area.

**Results:**

Weight-for-age *Z*-score (WAZ) and mid-upper arm circumference (MUAC) were independently associated with near-term mortality. The lowest WAZ threshold that, in combination with MUAC, detected all deaths associated with severe wasting or concurrent wasting and stunting was WAZ <−2·8. Performance for detecting deaths was best when only WAZ and MUAC were used. Additional criteria did not improve performance. Risk ratios for near-term death in children identified using WAZ and MUAC suggest that children identified by WAZ <−2·8 but with MUAC≥115 mm may require lower-intensity treatment than children identified using MUAC <115 mm.

**Conclusions:**

A combination of MUAC and WAZ detected all near-term deaths associated with severe anthropometric deficits including concurrent wasting and stunting. Therapeutic feeding programmes may achieve higher impact if WAZ and MUAC admission criteria are used.

Wasting and stunting are common conditions in children. It is estimated that 52 million children are wasted, 16 million of whom are severely wasted, and 155 million children are stunted^(^ ^1^ ^)^. Wasting and stunting are implicated in the deaths of almost 2 million children annually and account for over 15 % of all disability-adjusted life years lost in young children^(^ ^2^ ^)^. These figures are likely to underestimate burden because the number of wasted children present in a population (i.e. prevalent cases) found from unadjusted cross-sectional prevalence estimates will miss many incident cases of an acute condition^(^ ^3^ ^)^.

Wasting and stunting in children tend to be addressed as separate issues in programmes, policy and research. Recent reviews have called this separation into question^(^ ^4^ ^–^ ^8^ ^)^. Wasting and stunting are often present in the same populations^(^ ^9^ ^)^ and there is evidence that the two conditions share underlying and immediate causal factors^(^ ^10^ ^,^ ^11^ ^)^. Investigation into whether there is a direct causal relationship between wasting and stunting is ongoing and a number of gaps in the evidence base have been identified^(^ ^12^ ^)^. There has also been recent recognition that children may be both stunted and wasted at the same time^(^ ^13^ ^–^ ^15^ ^)^. National estimates of the prevalence of concurrent wasting and stunting in children, calculated for eighty-four countries, range between 0 and 8 %, exceeding 5 % in nine countries^(^ ^16^ ^)^. The factors leading to this state of ‘concurrence’ are poorly understood but the available evidence indicates that considerable excess mortality is experienced by these children. The level of excess mortality is not explained by the severity of their wasting and stunting alone. This suggests a multiplicative effect on mortality of having both deficits^(^ ^17^ ^,^ ^18^ ^)^. The magnitude of the mortality risk associated with concurrent wasting and stunting is similar to that associated with severe acute malnutrition (SAM)^(^ ^19^ ^)^. This raises the question of whether these children should be included in therapeutic feeding programmes which historically have treated only children with SAM as currently defined by the WHO and UNICEF using three independent diagnostic criteria:

1. mid-upper arm circumference (MUAC) below 115 mm;
2. weight-for-height *Z*-score (WHZ) below −3·0; and
3. the presence of bilateral nutritional oedema.

The presence of one or more of these criteria is enough to warrant admission into a therapeutic feeding programme^(^ ^20^ ^)^.

To investigate whether all children with concurrent wasting and stunting (WaSt) require therapeutic feeding and to better understand whether multiple diagnostic criteria are needed to identify children with a high risk of death and in need of treatment, an analysis examining which anthropometric indices are independently associated with near-term mortality is likely to prove a useful approach. The data for this type of analysis should come from a community-based cohort study assessing the risk of death in a representative sample of untreated children. Studies collecting data for such an analysis are no longer possible. The development of community-based management of acute malnutrition (CMAM) programmes capable of delivering effective SAM treatment at high levels of coverage have made it unethical to leave high-risk children untreated. The aim of the present paper is to describe the results of an analysis examining the ability of different anthropometric case definitions to identify children at high risk of near-term death. The analysis was performed using an existing data set from a historical community-based cohort study that pre-dates the development of CMAM programme models. An earlier analysis of the same data set showed strong relationships between a variety of nutritional indicators and mortality, with the highest population-attributable fractions associated with MUAC and WAZ^(^ ^21^ ^)^.

## Methods

### Data sources

The analysis presented here is based on a data set collected in 1983 and 1984 in Niakhar, a rural area of the Fatick region of central Senegal. These data have been described in detail elsewhere^(^ ^22^ ^)^. An open cohort of 5751 children, comprising all children under 5 years of age living in thirty villages in the study area, was followed through time. Each child was visited up to four times at 6-month intervals in May and November of 1983 and 1984. Anthropometric measurements were taken at each visit. All measurements were taken by the research staff after careful training and standardisation^(^ ^22^ ^,^ ^23^ ^)^. Weight was measured to the nearest 10 g using beam scales (SECA France, Semur en Auxois, France). Length and height were measured to the nearest 1 mm using ‘Harpenden’ length/height measurement boards (Holtain Ltd, Crymych, UK). MUAC was measured to the nearest 1 mm using non-elastic (fibreglass) tapes. Survival was monitored for all children using a demographic surveillance system operating in the study villages that was established in 1962^(^ ^24^ ^)^. The ages of children at each visit were calculated using the dates of birth taken from this demographic surveillance system and the dates of measurement by research staff. Approval for the original study was obtained from the International Health Department, the Scientific Commission and the Director General of ORSTOM/IRD (the French Institute for Scientific Research Overseas/Research Institute for Development) in France and from the Ministry of Health, Department of Statistics, Census Bureau and ORANA (Office de Recherches sur l’Alimentation et la Nutrition Africaines) in Senegal. Fieldwork was supported by ORSTOM/IRD, ORANA and the European Union’s DG-XII directorate (grant number TDR-36). Participation in the study was voluntary. Oral informed consent was obtained from principal caregivers. The analysis presented here did not involve new data collection and was done using anonymised records; therefore, it did not require further ethical approval.

Height-for-age, weight-for-age and weight-for-height *Z*-scores (HAZ, WAZ and WHZ, respectively) were calculated using the WHO growth standards^(^ ^25^ ^)^. The outcome of interest was death within 183 d (i.e. 6 months) of anthropometric assessment. The analysis was intended to investigate the association between anthropometry and near-term mortality to inform the selection of case-finding and admission criteria for therapeutic feeding programmes. These programmes treat SAM in children aged 6–59 months with the same standard protocol using ready-to-use therapeutic foods. These children represent the vast majority of children currently treated in such programmes. Therefore, the current analysis used data only for children aged 6–59 months at the time of anthropometric assessment (*n* 5144). The units of analysis used in the analyses are the individual 6-month follow-up episodes (12 638 episodes with 304 deaths).

Data management and data analysis were performed using purpose-written R language (version 3.5.1) scripts managed using the R Analytic-Flow scientific workflow system (version 3.1.8).

### Association between anthropometry and mortality

Bivariate associations between different anthropometric variables (i.e. HAZ, WAZ, WHZ and MUAC) and death within 6 months of measurement were examined by estimating mean values for died and survived follow-up episodes using Student’s *t* test and the common language effect size (probability of superiority) statistic^(^ ^26^ ^)^, which estimates the probability that a value drawn at random from the survived group will be greater than a value drawn at random from the died group. The null (i.e. no difference) value for the common language effect size statistic is 0·5. Multiple logistic regression was used to distinguish between real and apparent (i.e. due to confounding) associations. All variables with significant associations in the bivariate analyses were entered into the model. Non-significant variables were removed using backwards stepwise elimination with the least significant variable (*P* >0·05) removed at each step.

### Association between anthropometric status and mortality

Bivariate associations between anthropometric case definitions and death within 6 months of measurement were examined by calculating risk ratios from two-by-two tables. Standard case definitions for severe undernutrition were used;

1. Severely stunted: HAZ < −3·0.
2. Severely underweight: WAZ < −3·0.
3. Severely wasted: WHZ < −3·0.
4. Severely low MUAC: MUAC < 115 mm.

An additional anthropometric case definition for being concurrently wasted and stunted (WaSt) was also used;

5. WaSt: WHZ < −2·0 and HAZ < −2·0.

Multiple logistic regression with death within 6 months of measurement as the outcome, as described above, was used to distinguish between real and apparent (i.e. due to confounding) associations.

### Using anthropometry to predict mortality

The possibility of identifying all, or nearly all, children with severe anthropometric deficits who are likely to die within 6 months of measurement using a combination of anthropometric case definitions was explored using four-dimensional Venn diagrams with sets defined by WAZ, WHZ, MUAC and WaSt case definitions^(^ ^27^ ^)^. WHZ < −3·0 and MUAC < 115 mm case definitions were used because these are commonly used as admission criteria for programmes treating SAM^(^ ^20^ ^)^. An appropriate threshold for a WAZ case definition was found by a directed search for the lowest WAZ threshold that, in combination with MUAC, detected all deaths associated with the WHZ and WaSt case definitions. A second analysis used MUAC < 125 mm instead of MUAC < 115 mm. This threshold is used in programmes integrating treatment of both severe and moderate acute malnutrition^(^ ^28^ ^)^. Sensitivity, specificity and Youden’s index:


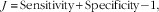


were estimated for case definitions based on combinations of WAZ, WHZ, MUAC and WaSt case definitions. Youden’s index measures overall performance of a screening or diagnostic test^(^ ^29^ ^)^. The maximum value of Youden’s index occurs for case definitions that optimise differentiating ability when equal weight is given to sensitivity and specificity. Risk ratios for death within 6 months of measurement in the populations selected using different combinations of MUAC and WAZ case definitions identified by the Venn diagram analysis were estimated.

### Effects of changing case definitions on programme caseloads

The effect of changing case definitions on program caseloads was investigated using simple ‘what-if?’ simulations. The model used was:

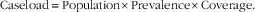




The population term was fixed at 17 000 assuming a service delivery unit (e.g. a health district) with a total population of 100 000 with 17 % of the total population aged between 6 and 59 months. Prevalence and coverage terms were modelled using fuzzy triangular numbers and fuzzy arithmetic with 95 % CI for ratio estimates calculated for a triangular distribution^(^ ^30^ ^,^ ^31^ ^)^. Prevalence was modelled using the 25th, 50th and 75th percentiles of prevalence estimates from 2426 nutritional anthropometry surveys from fifty-one countries from 1992 to 2015^(^ ^18^ ^)^. Coverage for programmes admitting using MUAC was modelled using the 25th, 50th and 75th percentiles of coverage estimates from 227 coverage assessments of CMAM programmes from twenty-nine countries from 2009 to 2017 recorded in a database provided by the Coverage Monitoring Network. Recent CMAM programme coverage estimates for cases meeting the WHZ < −3 admission criteria were unavailable. Work on coverage undertaken in pilots of the CMAM programme model found coverage of cases with WHZ < −3 to be consistently low (i.e. ≤20 %). It was assumed that coverage for WHZ cases would be one-third that of coverage for MUAC cases. It was assumed that cases with WAZ < −2·8 would be identified by growth monitoring or growth monitoring and promotion (GM/GMP) programmes. A literature review was undertaken and found useable coverage data for twenty-three GM/GMP programmes of differing scope and design from fourteen countries^(^ ^32^ ^–^ ^39^ ^)^. GM/GMP coverage was defined as a population coverage of more than three measurements in the previous 6 months. It was assumed that coverage in lower socio-economic groups (i.e. those groups most at risk of malnutrition) would be 80 % of reported coverage. It was also assumed that the interface between GM/GMP programmes and CMAM programmes would be imperfect, with 80 % of cases identified by the GM/GMP programmes being admitted into CMAM programmes. Coverage of children with WAZ < −2·8 was modelled using the 25th, 50th and 75th percentiles of the resulting estimates.

## Results

### Association between anthropometry and mortality

Table 1 shows the results of the analysis of bivariate associations between anthropometric variables and death within 6 months of measurement in the Niakhar cohort data. HAZ, WAZ, WHZ and MUAC were all negatively associated with death within 6 months of measurement. Table 2 shows the results of the multivariate analysis after non-significant variables had been removed. Only WAZ and MUAC were independently associated with death within 6 months of measurement.

**Table 1:** Bivariate associations between anthropometric variables and death within 6 months of measurement among children aged 6–59 months in the Niakhar cohort data, rural Senegal^(^ ^22^ ^)^

Variable	Outcome after 6 months	Mean	95 % CI	*P* ^*^	*w* †	95 % CI
HAZ	Died	−1·78	−1·92, −1·63	<0·0001	0·63	0·62, 0·64
	Survived	−1·18	−1·20, −1·16			
WAZ	Died	−2·35	−2·49, −2·20	<0·0001	0·71	0·70, 0·72
	Survived	−1·40	−1·42, −1·38			
WHZ	Died	−1·56	−1·72, −1·40	<0·0001	0·66	0·65, 0·67
	Survived	−0·79	−0·82, −0·77			
MUAC (mm)	Died	132·5	130·8, 134·1	<0·0001	0·70	0·69, 0·71
	Survived	143·4	143·1, 143·6			

HAZ, height-for-age *Z*-score; WAZ, weight-for-age *Z*-score; WHZ, weight-for-height *Z*-score; MUAC, mid-upper arm circumference.

* *P* value for Student’s *t* test.

† Common language effect size (probability of superiority) statistic. The statistic estimates the probability that a value drawn at random from the survived group will be greater than a value drawn at random from the died group. The null (i.e. no difference) value is 0·5. The statistic is based on comparison of *c*. 123 million pairs of values.

**Table 2:** Independent associations between anthropometric variables and death within 6 months of measurement^*^ among children aged 6–59 months in the Niakhar cohort data, rural Senegal^(^ ^22^ ^)^

Variable	OR	95 % CI	*P*
WAZ	0·75	0·64, 0·87	<0·0001
MUAC (mm)†	0·97	0·95, 0·98	<0·0001

WAZ, weight-for-age *Z*-score; MUAC, mid-upper arm circumference.

* Variables remaining in the model after non-significant variables were removed using backwards stepwise elimination.

† WAZ is recorded in *Z*-scores but MUAC is recorded in millimetres. A comparable OR for MUAC and death within 6 months of measurement is approximately 0·97^SD(MUAC)^ ≈ 0·97^13·72^ ≈ 0·66.

### Anthropometric status and mortality

Table 3 shows the results of the analysis of the bivariate associations between anthropometric case definitions and death within 6 months of measurement in the Niakhar cohort data. All anthropometric case definitions were associated with death within 6 months of measurement. Table 4 shows the results of the multivariate analysis after non-significant variables had been removed. Only severe underweight (WAZ < −3·0) and severely low MUAC (MUAC < 115 mm) were independently associated with death within 6 months of measurement.

**Table 3:** Anthropometric case status and relative risk of death within 6 months of measurement (bivariate analysis) among children aged 6–59 months in the Niakhar cohort data, rural Senegal^(^ ^22^ ^)^

Anthropometric status	Class	Case definition	RR	95 % CI	*P* ^*^
Stunted	Severe	HAZ < −3·0	2·79	2·10, 3·69	<0·0001
Underweight	Severe	WAZ < −3·0	3·97	3·13, 5·02	<0·0001
Wasted by WHZ	Severe	WHZ < −3·0	4·47	3·26, 6·12	<0·0001
Low MUAC	Severe	MUAC < 115 mm†	5·09	3·58, 7·23	<0·0001
WaSt	All	WHZ < −2·0 & HAZ < −2·0	4·08	3·13, 5·33	<0·0001

RR, risk ratio; WHZ, weight-for-height *Z*-score; MUAC, mid-upper arm circumference; WaSt, concurrent wasting and stunting; HAZ, height-for-age *Z*-score; WAZ, weight-for-age *Z*-score; CMAM, community management of acute malnutrition.

* *P* value for Fisher’s exact test.

† MUAC < 115 mm is commonly used for case finding in the community for admission to CMAM programmes. It is the primary CMAM programme admission criterion in many countries.

**Table 4:** Independent associations between anthropometric case status and death within 6 months of measurement^*^ among children aged 6–59 months in the Niakhar cohort data, rural Senegal^(^ ^22^ ^)^

Anthropometric status	Class	Case definition	OR	95 % CI	*P*
Underweight	Severe	WAZ < −3·0	3·51	2·65, 4·65	<0·0001
Low MUAC	Severe	MUAC < 115 mm†	2·20	1·41, 3·42	<0·0005

MUAC, mid-upper arm circumference; WAZ, weight-for-age *Z*-score; CMAM, community management of acute malnutrition.

* Variables remaining in the model after non-significant variables were removed using backwards stepwise elimination.

† MUAC < 115 mm is commonly used for case finding in the community for admission to CMAM programmes. It is the primary CMAM programme admission criterion in many countries.

### Using anthropometry to predict mortality

**Fig. 1:**
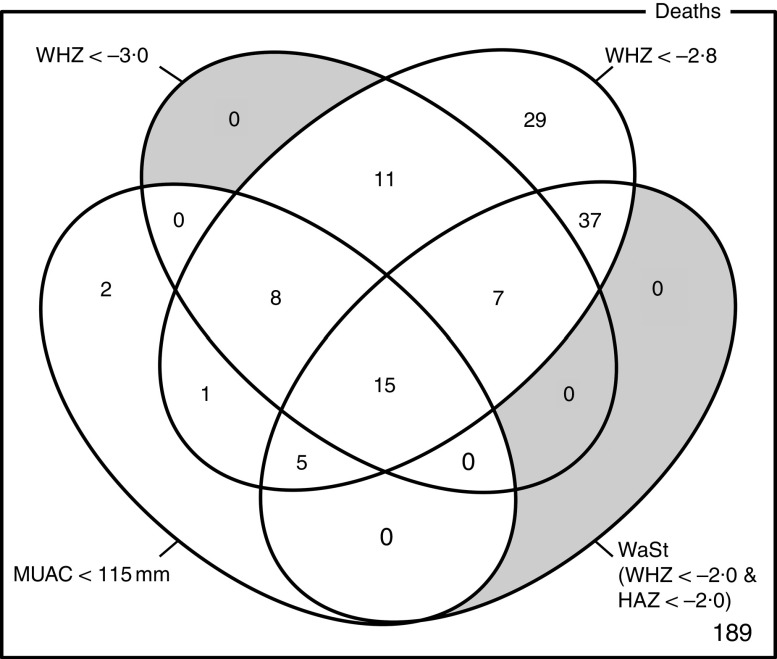
Numbers of deaths identified using MUAC < 115 mm, WHZ < −3·0, WAZ < −2·8 and WaSt case definitions among children aged 6–59 months in the Niakhar cohort data, rural Senegal^(^ ^22^ ^)^. The shaded area shows cells outside the union of the MUAC < 115 mm and WAZ < −2·8 sets. MUAC < 115 mm or WAZ < −2·8 detected all deaths associated with WaSt and with WHZ < −3·0. MUAC < 115 mm or WAZ < −2·8 detected more deaths than MUAC < 115 mm or WHZ < −3·0 (MUAC, mid-upper arm circumference; WHZ, weight-for-height *Z*-score; WAZ, weight-for-age *Z*-score; WaSt, concurrent wasting and stunting; HAZ, height-for-age *Z*-score)

**Fig. 2:**
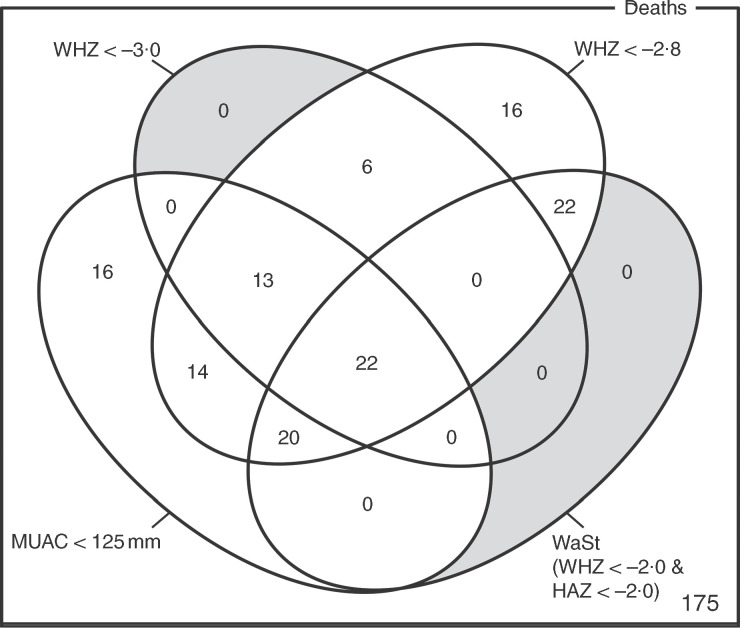
Numbers of deaths identified using MUAC < 125 mm, WHZ < −3·0, WAZ < −2·8 and WaSt case definitions among children aged 6–59 months in the Niakhar cohort data, rural Senegal^(^ ^22^ ^)^. The shaded area shows cells outside the union of the MUAC < 125 mm and WAZ < −2·8 sets. MUAC < 125 mm or WAZ < −2·8 detected all deaths associated with WaSt and with WHZ < −3·0. MUAC < 125 mm or WAZ < −2·8 detected more deaths than MUAC < 115 mm or WHZ < –3·0 (MUAC, mid-upper arm circumference; WHZ, weight-for-height *Z*-score; WAZ, weight-for-age *Z*-score; WaSt, concurrent wasting and stunting; HAZ, height-for-age *Z*-score)

The optimum WAZ threshold (i.e. the lowest WAZ threshold that detected all deaths associated with the WHZ and WaSt case definitions when used in combination with MUAC case definitions) was WAZ < −2·8. Figure 1 shows the four-dimensional Venn diagram for sets defined using the MUAC < 115 mm, WHZ < −3·0, WAZ < −2·8 and WaSt case definitions. The inclusion of WHZ < −3·0 and WaSt did not identify additional deaths when MUAC < 115 mm and WAZ < −2·8 were used to select high-risk children. Figure 2 shows the four-dimensional Venn diagram for sets defined using the MUAC < 125 mm, WHZ < −3·0, WAZ < −2·8 and WaSt case definitions. Increasing the MUAC cut-off to 125 mm identified additional deaths but WAZ < −2·8 still identified deaths not identified using MUAC.

Table 5 presents point estimates of sensitivity, specificity and Youden’s index for case definitions based on combinations of WAZ, WHZ, MUAC and WaSt case definitions. Overall performance (i.e. simplicity, the number of deaths detected, sensitivity, specificity and Youden’s index) was better for case definitions based on WAZ and MUAC. Adding WaSt or WHZ criteria resulted in slightly degraded performance (i.e. slightly reduced specificity).

**Table 5:** Point estimates of sensitivity, specificity and Youden’s index for detecting near-term deaths of different screening/admission criteria based on combinations of WAZ, WHZ, MUAC and WaSt case definitions among children aged 6–59 months in the Niakhar cohort data, rural Senegal^(^ ^22^ ^)^

Screening/admission criteria^*^	Deaths detected	Sensitivity	Specificity	Youden’s index
WaSt†	64	0·2105	0·9424	0·1529
WAZ < −2·8	113	0·3717	0·8704	0·2421
WaSt or WAZ < −2·8	113	0·3717	0·8675	0·2392
WHZ < −3·0	41	0·1349	0·9688	0·1037
WaSt or WHZ < −3·0	83	0·2730	0·9260	0·1990
WAZ < −2·8 or WHZ < −3·0	113	0·3717	0·8670	0·2387
WaSt or WAZ < −2·8 or WHZ < −3·0	113	0·3717	0·8640	0·2357
MUAC < 115 mm	31	0·1020	0·9801	0·0821
WaSt or MUAC < 115 mm	75	0·2467	0·9353	0·1820
WAZ < −2·8 or MUAC < 115 mm‡	115	0·3783	0·8677	0·2460
WaSt or WAZ < −2·8 or MUAC < 115 mm	115	0·3783	0·8648	0·2431
WHZ < −3·0 or MUAC < 115 mm	49	0·1612	0·9604	0·1215
WaSt or WHZ < −3·0 or MUAC < 115 mm	86	0·2829	0·9223	0·2052
WAZ < −2·8 or WHZ < −3·0 or MUAC < 115 mm	115	0·3783	0·8646	0·2429
WaSt or WAZ < −2·8 or WHZ < −3·0 or MUAC < 115 mm	115	0·3783	0·8617	0·2400
MUAC < 125 mm	85	0·2796	0·9180	0·1976
WaSt or MUAC < 125 mm	107	0·3520	0·8932	0·2452
WAZ < −2·8 or MUAC < 125 mm‡	129	0·4243	0·8455	0·2698
WaSt or WAZ < −2·8 or MUAC < 125 mm	129	0·4243	0·8427	0·2671
WHZ < −3·0 or MUAC < 125 mm	91	0·2993	0·9085	0·2079
WaSt or WHZ < −3·0 or MUAC < 125 mm	113	0·3717	0·8858	0·2576
WAZ < −2·8 or WHZ < −3·0 or MUAC < 125 mm	129	0·4243	0·8430	0·2674
WaSt or WAZ < −2·8 or WHZ < −3·0 or MUAC < 125 mm	129	0·4243	0·8403	0·2646

WAZ, weight-for-age *Z*-score; WHZ, weight-for-height *Z*-score; MUAC, mid-upper arm circumference; WaSt, concurrent wasting and stunting.

* The screening criteria (case definition) used to detect children with high risk of death.

† WaSt refers to WHZ < −2·0 and HAZ<−2·0.

‡ The best-performing (i.e. in terms of the number of indicators in the screening/admission criteria (fewer is better), the number of deaths detected (more is better) and Youden’s index (higher is better) screening criteria found in this analysis.

**Fig. 3:**
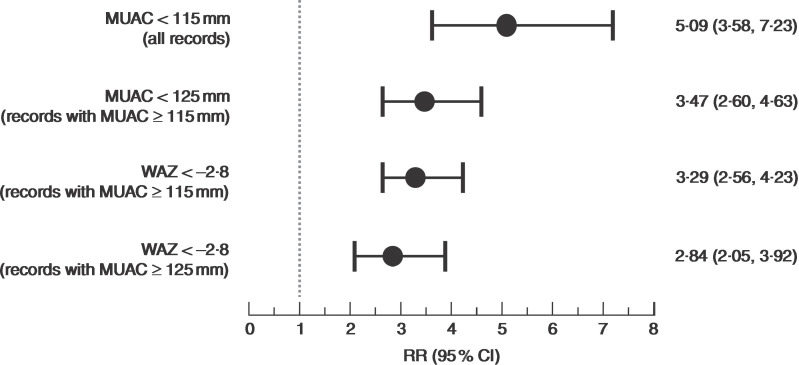
Risk ratios (RR) for death within 6 months of measurement (with their 95 % CI represented by horizontal bars) associated with different MUAC and/or WAZ case definitions among children aged 6–59 months in the Niakhar cohort data, rural Senegal^(^ ^22^ ^)^ (MUAC, mid-upper arm circumference; WAZ, weight-for-age *Z*-score

Risk ratios for death within 6 months of measurement in the populations selected using combinations of MUAC and WAZ case definitions identified by the Venn diagram analysis – i.e. MUAC < 115 mm; MUAC < 125 mm and MUAC ≥ 115 mm; WAZ < −2·8 and MUAC ≥ 115 mm; WAZ < −2·8 and MUAC ≥ 125 mm – are presented in Fig. 3.

### Effects of changing case definitions on programme caseloads

Table 6 presents the results of the simple ‘what-if?’ simulations of the effect of changing case definitions on programme caseload. The simulated caseload for programmes admitting using MUAC < 115 mm and WHZ < −3 was 123 (95 % CI 68, 260) cases per 17 000 population. The simulated caseload for programmes admitting using MUAC < 115 mm or WAZ < −2·8 was 355 (95 % CI 179, 644) cases per 17 000 population. The simulated caseload for programmes using MUAC < 115 mm or WAZ < −2·8 was 2·89 (95 % CI 1·38, 13·41) times larger than the simulated caseload for programmes admitting using MUAC < 115 mm or WHZ < −3. The simulated caseload for programmes admitting using MUAC < 115 mm only was 92 (95 % CI 50, 169) cases per 17 000 population. The simulated caseload for programmes admitting using MUAC < 125 mm only was 488 (95 % CI 300, 885) per 17 000 population. The simulated caseload for programmes using MUAC < 125 mm only was 5·30 (95 % CI 2·79, 25·54) times larger than the simulated caseload for programmes admitting using MUAC < 115 mm only.

**Table 6:** Results of the simple ‘what-if?’ simulations of the effect of changing case definitions on programme caseloads

Admission criteria	Population	Prevalence^*^ (%)	Coverage^*^ (%)	Caseload^*^
MUAC < 115 mm	17 000	(0·6, 1·3, 2·5)	(32·4, 41·6, 52·0)	(33, 92, 221)
MUAC < 125 mm		(4·2, 6·9, 11·1)		(231, 488, 981)
WHZ < −3 & MUAC ≥ 115 mm†		(0·7, 1·3, 2·4)	(10·8, 13·9, 17·3)	(13, 31, 71)
WAZ < −2·8 & MUAC ≥ 115 mm†		(4·1, 7.1, 10·9)	(12·5, 21·8, 26·8)	(87, 263, 497)

* Parameter values and results are presented as fuzzy triangular numbers.

† Cases additional to those found using the MUAC < 115 mm case definition.

## Discussion

The analysis presented here found that MUAC and WAZ were independently associated with death within 6 months of measurement. A previous multivariate (i.e. logistic regression) analysis of community-based cohort data from Bangladesh also reported that WHZ did not help identify high-risk children when MUAC was already in the model^(^ ^40^ ^)^.

A combination of MUAC and WAZ was able to detect all near-term deaths associated with severe anthropometric deficits including children who were simultaneously wasted and stunted (WaSt). This suggests that the MUAC admission criterion in therapeutic feeding programmes should be retained and that consideration should be given to replacing the current WHZ admission criterion with a WAZ admission criterion.

**Fig. 4:**
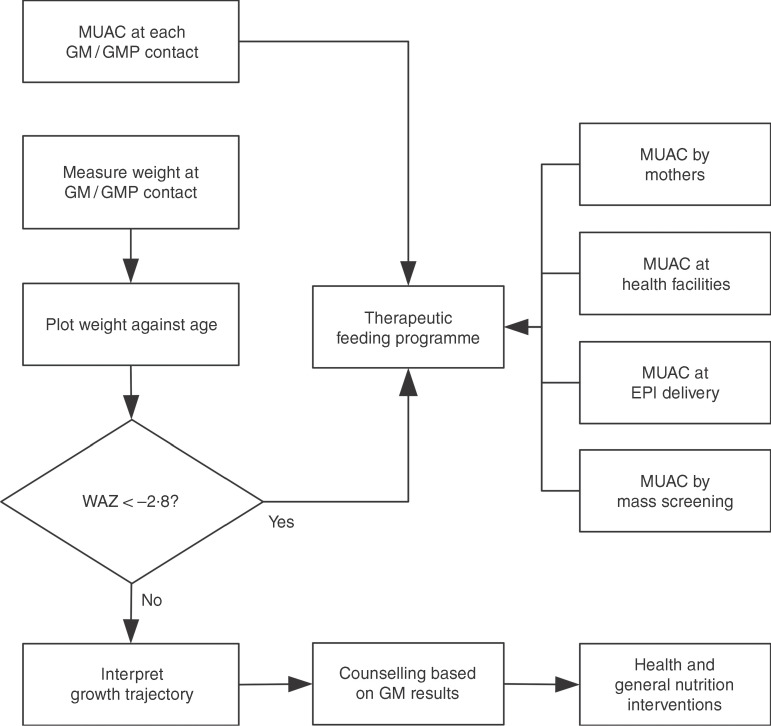
Delivery model of a programme linking GM/GMP and therapeutic feeding programmes enabling the use of MUAC and WAZ admission criteria in therapeutic feeding programmes (GM, growth monitoring; GMP, growth monitoring and promotion; MUAC, mid-upper arm circumference; WAZ, weight-for-age *Z*-score; EPI, expanded programme of immunisation)

WHZ < −3·0 was part of the clinical definition of severe malnutrition used in the 1999 WHO manual for the management of severe malnutrition^(^ ^41^ ^)^. This was based on the understanding that it reflected recent severe weight loss^(^ ^41^ ^)^. MUAC was introduced a decade later because it is easy to use in the community and is better at identifying children with a high risk of death than WHZ^(^ ^20^ ^,^ ^42^ ^)^. Using WHZ requires measurement of length or height. This is time-consuming, requires the presence of at least two health workers and requires length/height boards which are not available in many health facilities. Increasing the MUAC cut-off to 120 mm is likely to be more effective for identifying high-risk children than using the current combination of MUAC and WHZ^(^ ^43^ ^)^. If two anthropometric criteria are to be used to define severe undernutrition, it is not clear that WHZ in addition to MUAC is a good choice. Community-based cohort studies have repeatedly shown that WHZ is the least useful anthropometric criterion for identifying high-risk children and that WAZ performs significantly and consistently better at this task^(^ ^42^ ^–^ ^45^ ^)^. Recent analyses show that low WHZ is associated with a high risk of death only when it is also associated with stunting defined by low HAZ^(^ ^17^ ^,^ ^18^ ^)^. This is concurrent wasting and stunting (WaSt)^(^ ^18^ ^)^. Recent work on the descriptive epidemiology of multiple anthropometric deficits using data from almost 1·8 million children living in fifty-one countries reported that all children who were concurrently wasted and stunted were also underweight, the maximum possible WAZ for children with WaSt was below −2·35 when the WHO growth standards are used, and cases of WaSt can be detected with excellent sensitivity and specificity using WAZ^(^ ^18^ ^)^. This, together with the difficulty of providing good spatial and temporal coverage of the height measurements required for calculating HAZ and WHZ, means that WAZ may be used as a practicable indicator of WaSt. WAZ is easier to measure than WHZ because it does not require height or length to be measured. It does, however, lack the simplicity and low cost of MUAC. Any anthropometric indicator that includes an age component, such as WAZ, requires that age be ascertained accurately^(^ ^46^ ^)^. This is unlikely to be problem in GM/GMP programmes. Lack of simplicity, relatively high cost and the need to ascertain age accurately are likely to impair the utility of WAZ for detecting cases in the community^(^ ^42^ ^)^. Depending on the thresholds used, MUAC has higher attributable risks than WAZ^(^ ^21^ ^)^. This means that WAZ may be of most value when it is already being measured in (e.g.) GM/GMP programmes. Linkages between GM/GMP programmes and therapeutic feeding programmes could be forged and children with MUAC < 115 mm or WAZ < −2·8 referred to therapeutic feeding programmes from GM/GMP programmes (see Fig. 4).

Increases in caseload may not translate into similar increases in workload or costs if the additional cases identified by WAZ require lower-intensity treatment (e.g. smaller quantities of therapeutic feeding products and reduced frequency of contact) than cases identified by MUAC. A similar approach is used in programmes integrating treatment of both severe and moderate acute malnutrition^(^ ^28^ ^)^. Examination of mortality risks (Fig. 3) suggests that a low-intensity treatment may be appropriate for cases identified using WAZ. Simulation of caseloads for various scenarios (Table 6) suggests that the increase in caseload associated with replacing WHZ with WAZ is unlikely to be larger than that associated with moving from MUAC < 115 mm to MUAC < 125 mm which has proved to be manageable^(^ ^28^ ^)^.

Current programme designs tend to focus on either acute malnutrition (wasting) or chronic malnutrition (stunting). The distinction between acute and chronic malnutrition appears arbitrary^(^ ^4^ ^–^ ^11^ ^)^. An extensive literature review found no risk factor for wasting that was not also associated with stunting^(^ ^10^ ^)^. Being wasted is a risk factor for being stunted and vice versa^(^ ^18^ ^)^. The analysis presented here avoids the distinction between acute and chronic malnutrition and, following earlier work on admission criteria for CMAM programmes, focuses on near-term mortality risk associated with one or more anthropometric deficits^(^ ^42^ ^)^. Programmes admitting using MUAC and WAZ, both of which are associated with wasting and stunting, would treat children with anthropometric deficits associated with near-term mortality rather than just ‘acute malnutrition’.

Further work is required before the findings of the work reported here can be applied. This includes:

1. Repeating the current analysis using data from community-based cohort studies of untreated children from different contexts. This could further inform the selection of a usefully sensitive and specific (i.e. with regard to mortality) case defining WAZ threshold.
2. Ascertaining the intensity and duration of treatment required to treat children identified using WAZ as well as appropriate discharge criteria.
3. Operational research on linking GM/GMP and therapeutic feeding programmes.

The last two issues may be best addressed by small-scale (i.e. single health district) field studies. Such studies would also allow additional key programme planning data such as cost-effectiveness (e.g. cost per cure, cost per life saved, cost per disability-adjusted life year averted) and caseloads to be collected.

### Limitations

The analysis reported here uses data from a single study in a single location. Repeating the current analysis using data from community-based cohort studies of untreated children from different contexts would provide more compelling evidence. Nine additional cohort studies of similar design to the Niakhar study from Bangladesh, Ghana, Guinea Bissau, India, Indonesia, Nepal, Peru, Philippines and Sudan with archived data have been identified and efforts are underway to secure access to these data^(^ ^17^ ^,^ ^19^ ^,^ ^47^ ^–^ ^53^ ^)^. The caseload simulations are crude, and results should be treated as broadly indicative of the magnitude and direction of the effect of changing case definitions on programme caseloads.

## Conclusions

Therapeutic feeding programmes concerned with lowering the risk of near-term mortality may achieve higher impact if a combination of MUAC and WAZ admission criteria are used. More work is required before this can be considered as a general recommendation.
